# Twenty-year clinical experience with fixed functional appliances

**DOI:** 10.1590/2177-6709.23.2.087-109.sar

**Published:** 2018

**Authors:** Alexandre Moro, Suellen W. Borges, Paula Porto Spada, Nathaly D. Morais, Gisele Maria Correr, Cauby M. Chaves, Lucia H. S. Cevidanes

**Affiliations:** 1 Universidade Federal do Paraná, Programa de Pós-graduação em Ortodontia (Curitiba/PR, Brazil).; 2 Universidade Positivo, Programa de Mestrado e Doutorado em Odontologia Clínica (Curitiba/PR, Brazil).; 3 Mestre em Odontologia Clínica, Universidade Positivo (Curitiba/PR, Brazil).; 4 Universidade Federal do Ceará, Faculdade de Farmácia, Odontologia e Enfermagem (Fortaleza/CE, Brazil).; 5 University of Michigan, School of Dentistry, Orthodontics and Pediatric Dentistry (Ann Arbor, EUA).

**Keywords:** Orthodontics, Class II malocclusion, Fixed functional appliance.

## Abstract

**Introduction:**

Considering the large number of fixed functional appliances, choosing the best device for your patient is not an easy task.

**Objective:**

To describe the development of fixed functional appliances as well as our 20-year experience working with them.

**Methods:**

Fixed functional appliances are grouped into flexible, rigid and hybrid. They are different appliances, whose action is described here. Four clinical cases will be reported with a view to illustrating the different appliances.

**Conclusions:**

Rigid fixed functional appliances provide better skeletal results than flexible and hybrid ones. Flexible and hybrid appliances have similar effects to those produced by Class II elastics. They ultimately correct Class II with dentoalveolar changes. From a biomechanical standpoint, fixed functional appliances are more recommended to treat Class II in dolichofacial patients, in comparison to Class II elastics.

## RESUMO

**Introdução:**

considerando-se o grande número de aparelhos propulsores mandibulares, não é uma tarefa fácil escolher o melhor deles para o seu paciente.

**Objetivo:**

descrever o desenvolvimento desses aparelhos e a experiência clínica de vinte anos dos autores na sua utilização.

**Métodos:**

os aparelhos funcionais fixos aqui apresentados foram classificados em flexíveis, rígidos e híbridos, e o modo de ação de cada um deles foi descrito e ilustrado por meio de quatro casos clínicos.

**Conclusões:**

os aparelhos propulsores rígidos fornecem mais resultados esqueléticos do que os flexíveis e os híbridos. Esses últimos têm efeito semelhante ao uso de elásticos com direção de Classe II e, basicamente, corrigem a má oclusão de Classe II com alterações dentoalveolares. Do ponto de vista biomecânico, os propulsores fixos estão mais indicados para tratar a Classe II em pacientes dolicofaciais do que os elásticos de Classe II.

## INTRODUCTION

An arduous task in orthodontists’ daily routine is to convince adolescents or even adult patients to use Class II elastics or removable appliances. However, this is not new, since when Emil Herbst introduced the appliance he designed in 1909, Class II was corrected in Germany with the aid of a removable splint with inclined plane aimed at moving the mandible forward.^1^ Patients often did not comply with the use of removable appliances, thus, Herbst developed an appliance with a view to permanently moving the mandible forward with the aid of a fixed appliance, regardless of patient’s compliance.

After 1930, the Herbst appliance was seldom used, being rediscovered by Pancherz in 1979.^2^ After the Herbst appliance reappeared, more than 40 different fixed appliances aimed at correcting Class II malocclusion were developed in the last few years. In most cases, springs or metal tubes in combination with springs were used in order to replace the Herbst appliance telescopic system.

There is a wide range of fixed functional appliances available. Hence, choosing the best option for the patient is not an easy task. The present article aims at describing the development of fixed functional appliances as well as our 20-year experience working with them.

## CLASSIFICATION

To date, there is some doubt whether we should name appliances used to correct Class II malocclusion as mandibular protraction appliances or Class II correctors. Some appliances, also known as passive ones, result in mandibular protraction - in other words, move the condyle from the mandibular fossa, and the patient permanently bites with the mandible in forward position. Other appliances, also known as active ones, have a spring system that pushes the mandible every time the patient closes his/her mouth. However, it does so without moving the condyle from the fossa. In this case, they do not advance the mandible.

We particularly prefer Ritto e Ferreira's classification,^3^ which groups appliances according to the system of forces they use in order to move the mandible forward. Thus, appliances are grouped into: flexible, rigid or hybrid.

### Flexible fixed functional appliances

Flexible appliances are described as consisting of an intermaxillary coil spring or a fixed spring.^3^ Elasticity and flexibility are typical of those appliances. They allow for satisfactory free mandibular movement, with lateral guidance being easily performed. The amount of force varies and can be controlled by the clinician.

Their major drawback is the likelihood of both appliance and supporting system fractures (especially in the mandible). On one hand, flexibility is an advantage; on the other hand, it really tends to produce fatigue of springs. It is important to advise patients to avoid opening their mouths too widely because this could result in breakage. Additionally, they are not very esthetic appliances. If spring curvature is considerable, protuberances may appear in patient’s cheeks. 

Examples of flexible appliances include: Jasper Jumper,^4^ CS2000, and Jasper Vector.

### Rigid fixed functional appliances

These appliances are different from flexible ones in two respects: they are not easily fractured, however, they are not elastic nor flexible; after fitting and activation, they do not allow the patient to bite in maximal intercuspation as usual.^3^ This means the mandible is in forward position 24 hours a day, thereby providing more stimulus for growth. This group really results in mandibular protraction.

Rigid appliances work on the basis of a telescopic mechanism stimulating anterior repositioning of the mandible while the patient bites in occlusion. Skeletal effects produced by this appliance are greater than those produced by flexible ones. They are well described in the literature and will be discussed later in this article.

Examples of rigid appliances include: Herbst,^2^ MPA,^5^ and MARA.

### Herbst appliance

The Herbst appliance uses a bilateral telescopic system consisting of push rod and tube. It aims at permanently moving the mandible forward. As a result, muscles responsible for mandibular retrusion produce distalization force over maxillary teeth, while mesial force is produced against the mandible.

The Herbst appliance is probably the functional appliance most often used worldwide for correcting mandibular retrognathism. Despite not being a therapeutic unanimity, should diagnosis and patient selection be properly achieved, the appliance is able to successfully treat difficult Class II malocclusion cases in daily orthodontic practice. This even applies to non-compliant patients.

#### Herbst appliance design evolution

When Pancherz^2^ reintroduced the Herbst appliance, he used bands to manufacture it. In the 1990s,^6^ bands were replaced by metal splints made of a chromium-cobalt alloy bonded to teeth with glass ionomer. The system^6^ ensured accurate fit to teeth, in addition to being resistant and hygienic, shortening chair time, and causing little clinical trouble. Nevertheless, this new design increased costs for appliance manufacture.

From 1982 onwards, Howe^7^ and McNamara Jr.^8^ began developing the Herbst appliance with acrylic splint. Initially, splints were bonded to patients’ dental arches. However, they noticed that the Herbst appliance with splints bonded to maxilla and mandible involved a risk to the patient. This was because teeth were most likely to decalcify under such conditions, which also led to caries and enamel fracture at appliance debonding. Nowadays, the model is seldom used.

The Herbst appliance consisting of stainless-steel crowns bonded to maxillary first molars and an acrylic splint covering the occlusal surface of mandibular teeth was introduced in 1989.^9^ The system allowed the mandibular piece to be temporarily removed, thus making oral hygiene and adjustment to under-eruption teeth easier. 

In 1994, Mayes^10^ introduced the Cantilever Bite Jumper (CBJ). This appliance consisted of four stainless-steel crowns bonded to maxillary and mandibular first molars associated with a cantilever welded to mandibular first molar crowns, which extended anteriorly to the premolar and canine area, where the mandibular pivot was placed. Design advantages included allowing for use in mixed dentition without the need for premolar bands.

**Figure 1:**
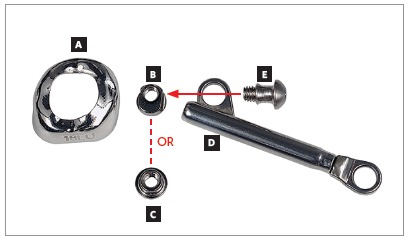
Miniscope telescopic system: A) Rollo band; B) Universal nut; C) Barrel nut; D) Miniscope (right side); E) Applecore screw.

For many years, Ormco stainless-steel crowns (Orange, CA, USA) remained the best option for Herbst appliance manufacture. These crowns were highly resistant, however, debonding was an arduous task. To date, bands most widely used for Herbst appliance manufacture are Rollo bands (American Orthodontics, Sheboygan, WI, USA). Their occlusal surface is partially coated, they have retention similar to a crown, and the versatility of bands (Fig 1). 

#### Types of telescopic systems

Herbst appliance design was modified over the years. Similarly, the telescopic system evolved, so as to provide more resistance to the appliance, prevent fractures,^11,12^ and bring comfort, thus enhancing patient’s adaptation. 

The most important models are:

» Dentaurum types I, II, IV and TS (Dentaurum, Ispringen, Germany).
» Flip - Lock (TP Orthodontics, La Porte, IN, USA).
» Hanks-HTH and Miniscope (Fig 1) (American Orthodontics, Sheboygan, WI, USA).
» Abzil Mandibular Protraction Appliance, PMA (3M - ABZIL, São José do Rio Preto, SP, Brazil).
» AdvanSync (Ormco, Orange, CA, USA).
» M4 (Specialty Apliances, Cumming, GA, USA).
» Manni Telescopic Herbst (MTH) (Micerium, Avegno, GE, Italy).

#### What are the dentoskeletal effects produced by Herbst appliance?

When Class II malocclusion with 6-mm molar relationship is corrected with the aid of Herbst appliance, correction may result from several sources, namely: restricted maxillary growth; increased mandibular growth; maxillary molars distalization; mandibular molars mesialization. The level of contribution provided by each one of those sources depends not only on appliance design, but also on patient’s growth stage. 

A number of Herbst designs have been developed, especially with a view to avoiding mesialization of mandibular teeth; nevertheless, even an increased number of teeth involved with mandibular appliance anchorage did not prevent it. Fixed appliance assembly in the mandible during use of Herbst appliance further increased proclination of mandibular incisors.^13^ In 2009, Martin and Pancherz^14^ found an association between the amount of forward mandibular movement at treatment onset and proclination of incisors. The greater forward mandibular movement at treatment onset, the greater the intrusion, protrusion and anterior tipping of mandibular incisors. However, the authors^14^ highlighted that during the next phase involving use of fixed appliance, the aforementioned movements were reversed. Further on making a significant forward mandibular advancement at treatment onset or step-by-step forward movement, a few studies^15^ suggest better mandibular growth response as a result of step-by-step forward movement, whereas other studies^16^ show no difference. Nevertheless, many clinicians opt for appliance use during 6 to 8 months only, and prefer significant forward mandibular movement at treatment onset, since the time available for the step-by-step procedure would be limited.

As regards mandibular growth, generally speaking, it is possible to claim that one year using the Herbst appliance will allow patient’s mandible to grow an average of 1.3 to 1.7 mm more in comparison to non-use of the appliance.^17^ ^,^ ^18^ In a systematic review on mandibular changes produced by functional appliances, Cozza et al^19^ concluded that the Herbst appliance showed the highest coefficient of efficiency.

It is important to highlight that mandibular growth can be clinically stimulated; however, not in all Class II patients. Patients with the best responses^20^ are those having gonial angle around 122°. Dolichofacial patients do not present satisfactory growth response.^21^

**Figure 2:**
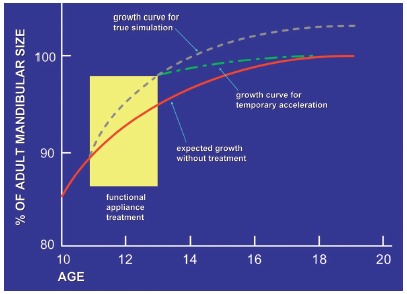
An illustration of true stimulation and temporary stimulation of mandibular growth. True stimulation indicates that growth occurs at a faster-than-expected rate during functional appliance therapy, then continues at the expected rate thereafter, so that the ultimate size of the mandible is larger. Temporary acceleration means that faster growth occurs during functional therapy, but slower growth thereafter ultimately brings the mandible back to the size that would be expected without treatment (Adapted from:Lai and McNamara^23^, 1998).

Presently, a major controversy over functional appliances is whether the gain they provide lasts in the long-term. Some researchers believe it does;^22^ however, others claim the appliances only speed up and/or antedate mandibular growth during appliance use. After removal, mandibular growth decreases and reaches the same size as if no appliance had been used^23^ ^,^ ^24^ (Fig 2). It is worth highlighting there is lack of systematic reviews and meta-analyses based on stronger evidence dealing with the appliance long-term effects.

#### 3D studies

Nearly all research on Herbst appliance effects results from studies based on cephalograms in lateral view. The latter have their limitations and lead to doubt over the reliability of studies.^25^

**Figure 3:**
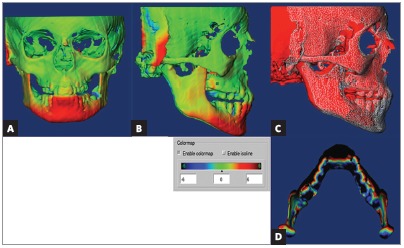
Tridimensional assessment carried out by means of colored maps after one year of treatment with the Herbst appliance: A) frontal view; B) lateral view; C) lateral view of mesh superimposition; D) mandibular occlusal view.

Using advanced techniques consisting of 3D imaging and superimposition overcomes such limitations, as they allow for accurate analysis and measurement of changed position not only in the maxilla, but also in the mandible relative to the cranial base (Fig 3).

LeCornu et al^26^ concluded that patients treated by means of Herbst appliance presented with forward displacement of condyles and fossa along with restricted maxillary growth, when compared to a control group. Borges et al^27^ assessed patients with initial mean age of 9 years and who were subjected to treatment with Herbst appliance. The authors found that remodeling at TMJ joint region was insignificant and lower than 1 mm. There was no stimulus for mandibular growth provided by the appliance. Souki et al^28^ assessed patients aged from 12 to 16 years old, and found that significant forward displacement of the mandible was achieved as a result of increased bone remodeling in condyles and rami, when compared to a control group. Furthermore, 3D changes in direction and degree of condylar growth were found in patients treated with the Herbst appliance.

#### What is the best moment to use the Herbst appliance?

Several studies have shown the best moment to try stimulating mandibular growth with the aid of the appliance is right before reaching the peak in pubertal growth spurt.^29^

Nevertheless, Behrents^30^ published an editorial reporting up-to-date safe scientific evidence suggesting the early Class II treatment onset in cases with patients presenting protruding maxillary incisors. Treatment is justified because decreased protrusion protects incisors against trauma, in addition to enhancing patient’s self-confidence and social adjustment. 

#### When should the appliance be removed?

**Figure 4:**
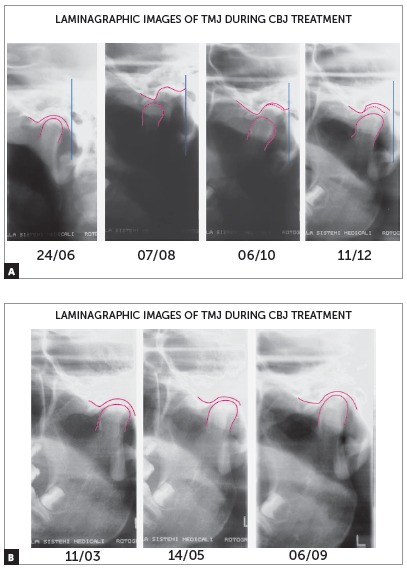
TMJ laminagraphic images of patient treated with Cantilever Bite Jumper (CBJ). A) Before treatment onset, condyles were centered in the fossa. At appliance placement, the mandible was moved 9mm (07/08) forward. Two and four months later, double condyle and mandibular fossa images are seen as a result of bone remodeling. B) Seven months later (11/03), the condyle was back to its primary position.

The appliance is typically used within a period of 8 to 12 months. Condyles must be centered in the mandibular fossa at the time of removal (Fig 4). Additionally, one should always consider patients will suffer a relapse in terms of dental relationship, thus, it is paramount to overcorrect molar relationship and, if possible, reach Class III. After the appliance has been removed, fixed appliance should be assembled in order to achieve a perfect detailing of the occlusion. 

Although some studies^31^ show that the condyle is back to its initial position in the mandibular fossa after eight months, we prefer to use the appliance for twelve months, to ensure there will not be relapse in condyle and mandibular fossa remodeling. A number of studies have shown that the duration of forward movement is a critical factor in maturation of newly formed bone and stability of outcomes.^32^ ^,^ ^33^ Late appliance removal might prevent little growth and increase maturation of newly formed bone matrix to the same degree of bone formed during development and bone repair.^32^ Studies^32^ have shown that a 6-month period is required for newly formed bone (former collagen matrix type III) to mature into collagen matrix type I, with the latter being more stable. Therefore, mandibular advancement is necessary, and so it is keeping the mandible in forward position for at least six months.^34^ Tomblyn et al^33^ have recently suggested that the Herbst appliance should be used for 18 months; however, total Class II treatment time might take too long, especially if we consider that the second phase of treatment by means of fixed appliance lasts between 12 and 24 months.

## CLINICAL CASE 1 (Figs 5 to 10)

Patient aged 11 years and 5 months old at treatment onset. At the time, diagnosis was of Class II, division 1, with 3.0-mm overbite and 8.0-mm overjet. The patient was near the peak of pubertal growth spurt. Due to mandibular retrusion, the option was for moving the mandible forward with the aid of Herbst appliance. The latter consisted of stainless-steel crowns on maxillary first molars and mandibular removable splint.^35^ Seven months later, fixed appliance was assembled onto the maxilla with a view to aligning and leveling teeth. The Herbst appliance was removed one year after its placement. Overcorrected Class I molar relationship was achieved. Thereafter, appliance was placed onto the mandible with a view to enhancing patient’s occlusion. Treatment lasted for 36 months. Ten years after treatment conclusion, the patient presented with good stability of correction achieved. 

**Figure 5:**
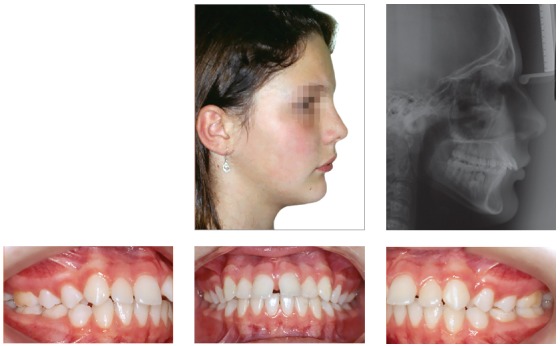
Pretreatment records: Extra and intraoral photographs, and lateral cephalogram.

**Figure 6:**
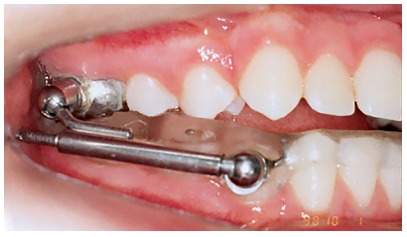
Intraoral right photograph showing Herbst appliance placement.

**Figure 7:**
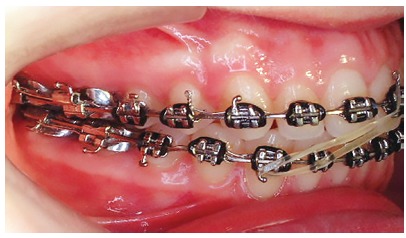
Intraoral right photograph showing fixed appliance.

**Figure 8:**
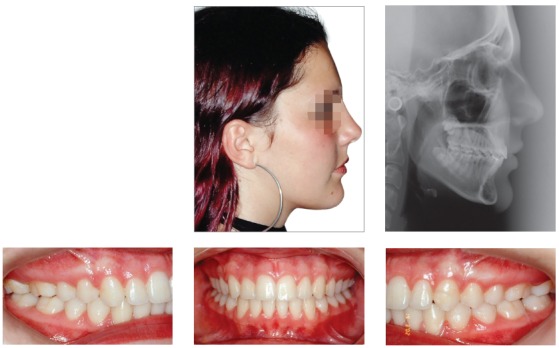
Posttreatment records: Extra and intraoral photographs, and lateral cephalogram.

**Figure 9:**
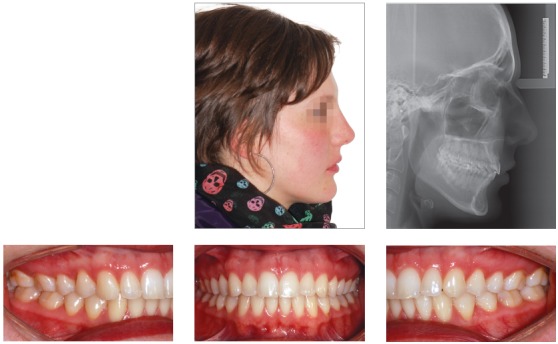
Posttreatment records at ten years: Extra and intraoral photographs, and lateral cephalogram.

**Figure 10:**
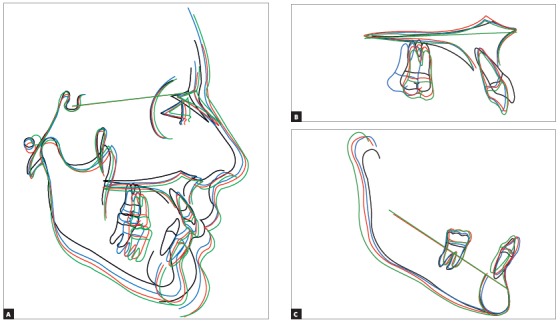
A) Cephalometric tracings superimposition on the cranial base (black = initial; blue = after Herbst appliance; red = treatment completion; green = ten years after treatment completion). B) Maxillary superimposition (ANS-PNS registered at ANS). C) Mandibular superimposition (Xi-Pm registered at Pm).

## CLINICAL CASE 2 (Figs 11 to 16)

Patient aged 11 years and 3 months old at treatment onset. At the time, diagnosis was of Class II, division 1, with 8.0-mm overbite and 5.0-mm overjet. The patient was near the peak in pubertal growth spurt. Maxilla was well positioned, while mandible was retruded. Due to significant lack of space in the maxilla, rapid maxillary expansion was initially carried out. One month after screw immobilization, maxillary fixed appliance was placed with a view to leveling maxillary incisors. Thereafter, for Class II correction, the option was for moving the mandible forward with the aid of Herbst appliance. The latter consisted of steel crowns on both maxillary and mandibular first molars, in addition to cantilever aimed at providing support to mandibular pivots. The Herbst appliance was removed one year after its placement. Overcorrected Class I molar relationship was achieved. Thereafter, appliance was placed onto the mandible in order to enhancing patient’s occlusion. Treatment lasted for 35 months. Five years and six months after treatment conclusion, the patient presented with good stability of the correction achieved. 

**Figure 11:**
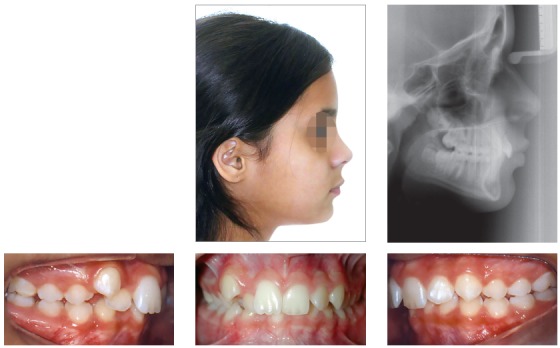
Pretreatment records: Extra and intraoral photographs, and lateral cephalogram.

**Figure 12:**
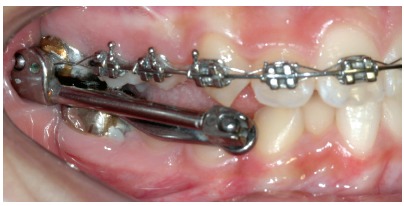
Intraoral right photograph after Herbst appliance placement.

**Figure 13:**
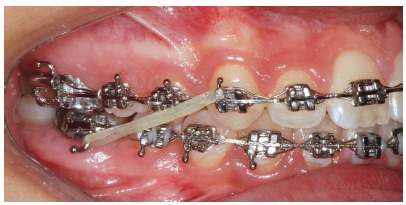
Intraoral right photograph showing fixed appliance.

**Figure 14:**
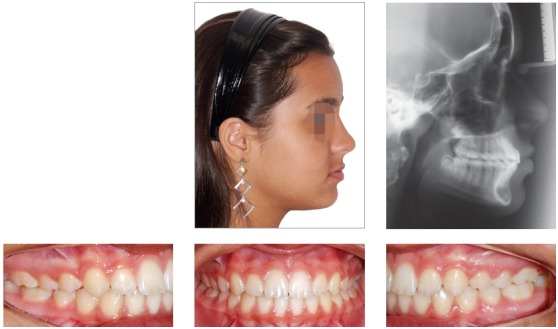
Posttreatment records: Extra and intraoral photographs, and lateral cephalogram.

**Figure 15:**
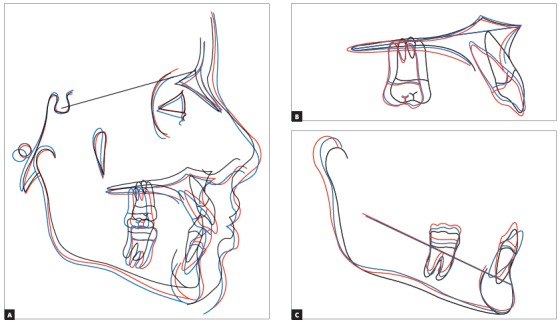
A) Cephalometric tracings superimposition on the cranial base (black = initial; blue = after Herbst appliance; red = treatment completion). B) Maxillary superimposition (ANS-PNS registered at ANS). C) Mandibular superimposition (Xi-Pm registered at Pm).

**Figure 16:**
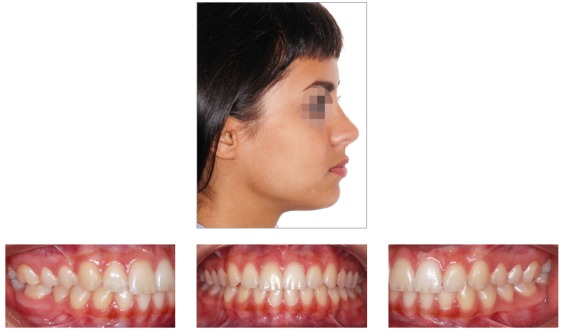
Posttreatment records at 5.5 years: Extra and intraoral photographs.

### Hybrid fixed functional appliances

Hybrid appliances are a combination of flexible and rigid ones. They are rigid appliances with spring systems.^3^ The purpose of these appliances is to move teeth by applying continuous elastic force 24 hours a day. This replaces conventional Class II elastics. Use of open springs to produce force is typical of this type of appliance. Force produced varies from 150 to 260g. The main purpose of hybrid appliances is not to reposition mandible in forward position. Based on the literature,^36^ ^,^ ^37^ it is possible to claim that flexible and hybrid appliances produce greater tooth movement during treatment, in comparison to rigid ones. This is probably due to not moving the condyle from the mandibular fossa. Examples of hybrid appliances include: Forsus, Twin Force, Sabbagh Universal Spring (SUS), and PowerScope.

Hybrid functional appliances have been widely used in the last few years, particularly due to Forsus considerable success. A number of hybrid appliances have been developed in an attempt to outdo Forsus. The following characteristics are typical of this new generation of appliances: spring inserted into the telescopic system, to avoid hurting patient’s cheek and prevent food from accumulating during meals;^38^ reduced size, to provide more comfort and favor patient’s adaptation.

#### Forsus

**Figure 17:**
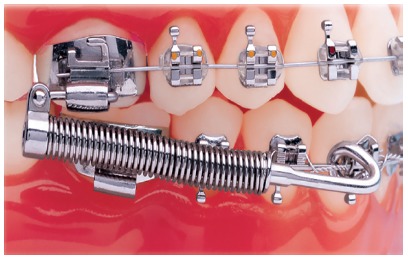
Forsus Fatigue Resistant Device with L-pin Module, released in 2002.

**Figure 18:**
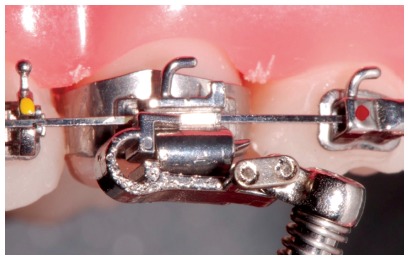
Forsus Fatigue Resistant Device with EZ2 Module. Note two screws on maxillary molar clip (modification released in 2009).

**Figure 19:**
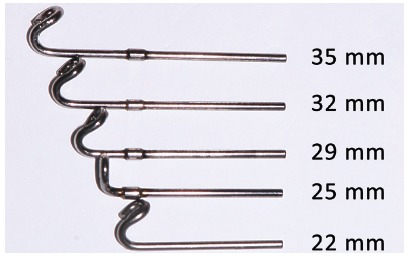
Forsus appliance push rods in different sizes: extra short push rod = 22 mm; short push rod = 25 mm; median push rod: 29 mm; large push rod = 32 mm; extralarge push rod = 35 mm.

Forsus appliance (Fig 17) consists of three pieces:^39^ Spring - which is fatigue resistant, made of stainless steel, and produces force of approximately 220g; Clip - appliance piece aimed at securing the spring into maxillary molar tube (Fig 18), it has an anti-rotational stop used to provide stability, thus preventing the appliance from moving during use (in the middle of the clip, there is a space used to attach the headgear tube); Push rod - appliance piece that connects the appliance to the mandible (at its lower end, a loop locks it onto the lower arch; right before the loop, there is a raised piece consisting of a spring stop, Fig 19).

**Figure 20:**
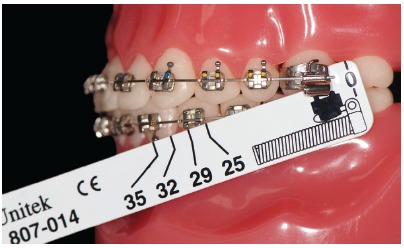
Measurement gauge placement. The tool is used to choose the size of Forsus appliance. With the patient biting and having the mandible in maximal intercuspation, the clinician places the buccal portion of the measurement gauge behind the maxillary molar tube. The tool is then tipped and the number near the distal portion of the canine bracket or mandibular first premolar is chosen.

In order to best choose the appliance, an appropriate measurement gauge is necessary (Fig 20).

**Figure 21:**
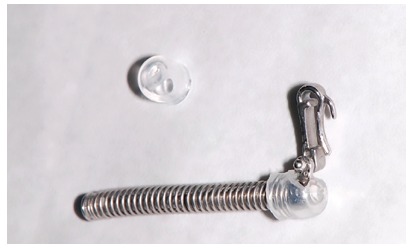
Plastic caps used to protect patient’s cheek when Forsus appliance is used.

It is rather common that with the use of Forsus appliance, the patient feel some discomfort or cheek irritation during the first days. Patient may use utility wax or Comfort Solutions plastic caps for protection (Fig 21). 

#### General clinical requirements for installation

Similarly to all mandibular protraction appliances, Forsus tends to protrude mandibular teeth. Thus, the ideal is to reinforce mandibular anchorage. Therefore, a 0.019 x 0.025-in stainless steel archwire must be used with 0.022-in slot, or 0.017 x 0.025-in stainless steel archwire must be used with 0.018-in slot. With a view to avoiding protrusion of mandibular incisors, resistant lingual torque on mandibular teeth in the anterior region or brackets with greater lingual torque on those teeth should be considered. An Omega loop is also interesting to secure the archwire. A bend on the distal surface of last molar is also considered. It is also recommended to use a figure-8 stainless-steel ligature in all lower teeth, since the appliance tends to open space between canines and first premolars. The use of a lingual arch in the mandible and a transpalatal arch in the maxilla is recommended. A last requirement is the use of an occlusal headgear tube in the maxillary molar.^40^

## CLINICAL CASE 3 (Figs 22 to 28)

Patient aged 13 years at treatment onset. At the time, diagnosis was of Class II, division 1, with deep bite. Both maxilla and mandible were slightly protruded. There was dentoalveolar mandibular retrusion. Lower facial height was decreased. Overjet was 18mm. The mandible was not retruded, therefore, the option was for Class II correction with dentoalveolar changes. X-bow^41^ appliance was used. Initially, a Hyrax with extraoral tube on maxillary first molars was installed. One month after expansion appliance screw immobilization, a modified lingual arch with loop in the region of mandibular first premolars was installed to allow Forsus appliance push rod placement. Two months after X-bow was installed, a fixed appliance was placed in the maxilla. Seven months later, overcorrected Class I molar relationship was achieved. Subsequently, second treatment phase began with mandibular fixed appliance assembly. After leveling, maxillary anterior retraction was carried out with Bull loop. Once arch coordination and intercuspation had been achieved, the fixed appliance was removed and retainers installed: maxillary modified Hawley retainer and mandibular fixed 3x3 retainer. Treatment lasted for 30 months. Six years after treatment conclusion, the patient presented with good stability of correction achieved. 

**Figure 22:**
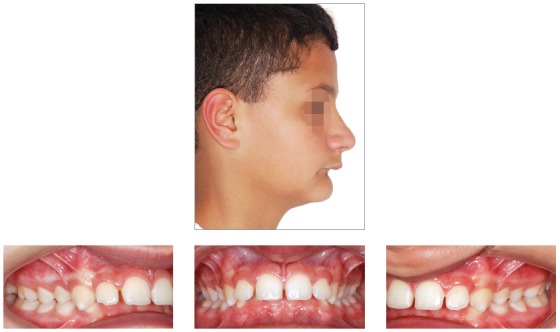
Pretreatment records: Extra and intraoral photographs.

**Figure 23:**
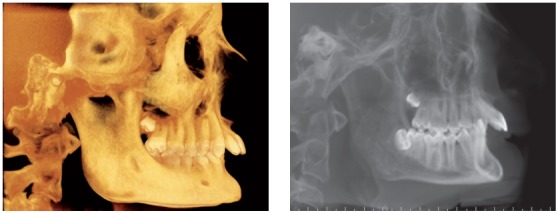
Initial tomographic scan in lateral view and lateral cephalogram.

**Figure 24:**
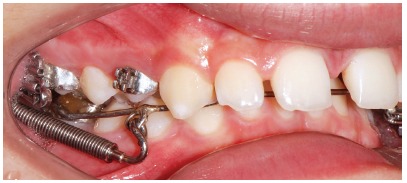
Intraoral photograph showing X-bow placement.

**Figure 25:**
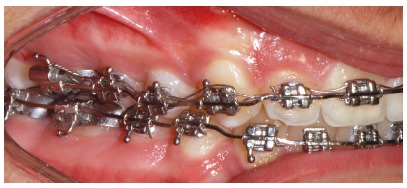
Intraoral photograph showing fixed appliance.

**Figure 26:**
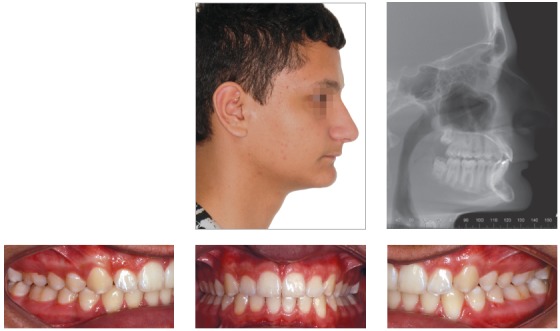
Posttreatment records: Extra and intraoral photographs, and lateral cephalogram.

**Figure 27:**
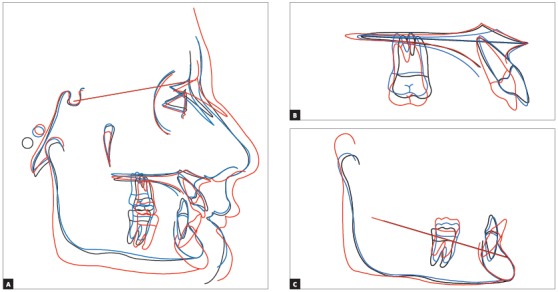
A) Cephalometric tracings superimposition on the cranial base (black = initial; blue = after X-bow appliance; red = treatment completion). B) Maxillary superimposition (ANS-PNS registered at ANS). C) Mandibular superimposition (Xi-Pm registered at Pm).

**Figure 28:**
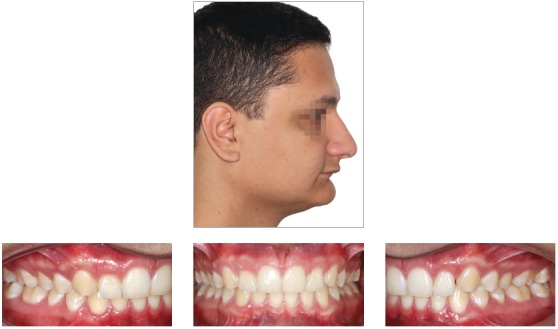
Posttreatment records at 5 years: Extra and intraoral photographs.

### Powerscope 2

PowerScope^42^ ^,^ ^43^ is a new generation of hybrid fixed functional appliance. Released in 2014, a year later it was subjected to three changes (stop reinforcement, magnet key, and activation indicator piece). As a result, the appliance was renamed PowerScope 2. 

**Figure 29:**
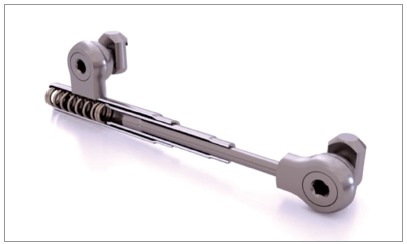
PowerScope appliance spring. The photograph shows how the spring remains inside the appliance.

**Figure 30:**
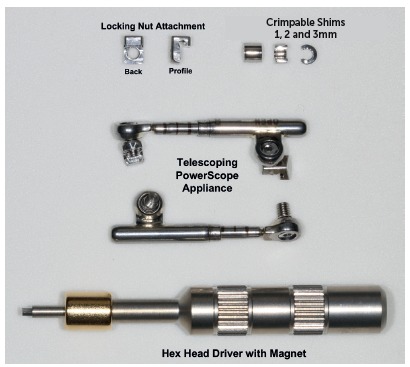
Components of PowerScope.

PowerScope appliance consists of a telescopic system with three fitting pieces that will not come loose during treatment. It comes as an one-size-fits-all appliance, which helps to control and save storage room. Its internal mechanism consists of a nickel-titanium spring producing 260-g force (Fig 29). Additionally, a wire-to-wire connection is also present, thus allowing for quick and easy installation. The appliance can be placed with tubes bonded to molars or tubes welded to bands. The telescopic system comprises attachments nuts with hex screws on their ends, with the former being responsible for securing the system onto the fixed appliance arch. To fasten the screw, an Allen hex key wrench is used. The tool has a magnet that aids appliance assembly (Fig 30). The attachment nut has a slot that is closed with the screw thread, so the screw forms a fourth surface (the inferior) to capture the wire when tightened. The slot that is formed is 0.020 x 0.026-in. Although it may seem that the attachment nut pinches the wire, in fact, it does not. In the maxilla, the system freely slides, thereby making molar distalization easier. In the mandible, the system also slides, but it does not end up reaching the canine bracket, due to arch curvature. As a result, bracket detachment decreases.

Since the appliance is placed in maxilla and mandible, stainless-steel archwires should be used in both of them. With 0.022-in slot, wire diameter must be 0.019 x 0.025-in; with 0.018-in slot, it must be 0.017 x 0.025-in. Previously described requirements for mandible preparation before Forsus placement also apply to PowerScope.

**Figure 31:**
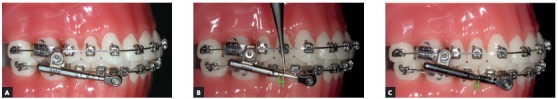
Evaluation of PowerScope activation: A) Appliance placed without activation, as evinced by three black lines; B) Mid tube being pushed backwards with a tool; C) 6-mm spacer placement for total spring activation. Note that the black lines disappear, while a 1.5-mm depression in intermediate tube appears (marked in green).

**Figure 32:**
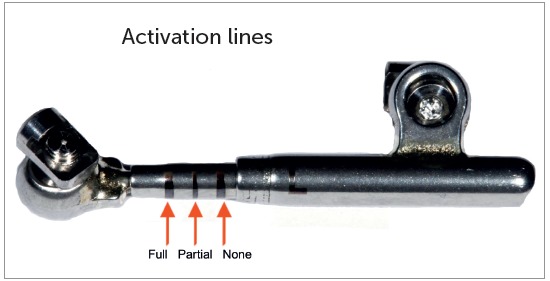
Activation indicator. Should three marks appear, this means the spring is not activated. Should two marks appear, this means the spring is partially activated. Should no marks appear, the appliance is totally activated.

Depending on the size of teeth and severity of Class II malocclusion, spacers might be necessary early during placement, to activate the appliance (Fig 31). With patient’s bite in maximal intercuspation as usual, the clinician is advised to push the middle tube backwards with the aid of any tool - for instance, a probe -, in order to check the amount of movement. That is the quantity to which appliance should be activated to allow the spring to remain completely compressed. Clinician should then place the appropriate spacer over the lower push rod until correct activation is achieved. The activation indicator (Fig 32) also helps assessing whether activation is correct or not. Whenever the patient comes back for the next appointment, the clinician should first ask him/her to bite in maximal intercuspation as usual, and then assess accordingly. 

**Figure 33:**
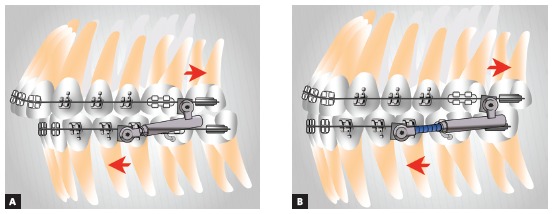
Use of spacers in mandibular push rod: A) No spacer; B) After placement of six 1-mm spacers.

We always work with PowerScope spring completely activated, regardless of performing 3-mm or 6-mm movement. During the following appointment one month later, tooth movement is usually noticed, and the patient will likely lose around 1-mm activation. Thus, we basically reactivate the spring every month until 1-mm or 2-mm overcorrection is achieved for the buccal segment (Fig 33).

### Step-by step installation

**Figure 34:**
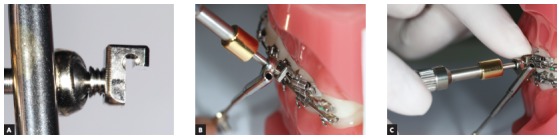
Step-by step placement: A) Initially, screw end should be leveled with the internal stop surface; B) Subsequently, the attachment nut is tipped in 45° relative to the arch; C) The nut should be pressed gently with clinician’s index finger, so as to fit the attachment nut into the arch and allow the appliance to remain parallel the occlusal plane. Thereafter, key should be turned with clinician’s right hand in short turns. Placement should be performed first in the maxilla and then in the mandible.

PowerScope was developed to be installed on maxillary first molar mesial surface and mandibular canine distal surface (Fig 34).

**Figure 35:**
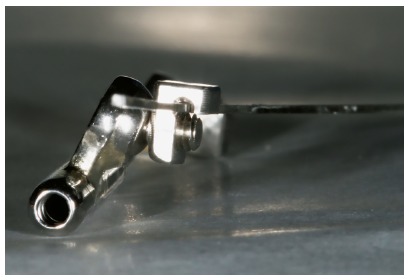
Appliance placement outside the mouth. Note the arch inside the attachment nut.

**Figure 36:**
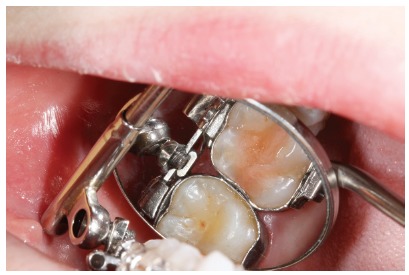
Mirror used to check whether the screw is completely closing the nut slot, thus preventing the attachment nut from falling loose.

The appliance can be installed outside of the mouth on the upper archwire (Fig 35). Thereafter, the upper archwire is placed into tubes and brackets. Subsequently, it is placed in the mandible. It is also paramount to assess whether the screw is in fact completely closing the slot, and whether the arch is placed inside it. Assessment can be achieved with the aid of a mirror (Fig 36). 

**Figure 37:**
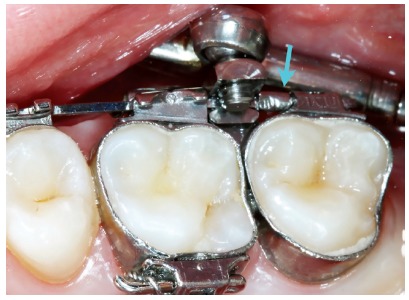
Stop placement on the mesial surface of maxillary second molar tube.

**Figure 38:**
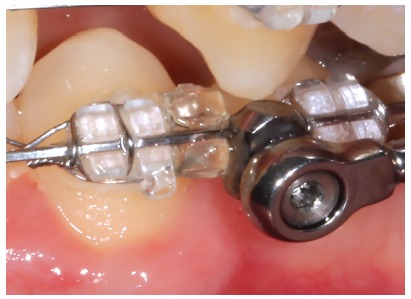
Elastomeric ligature (With Guard, 3M Unitek, Monrovia, CA, USA) placement on distal wing of mandibular first premolar bracket. A rotation edge can also be used.

The appliance can also be installed on mandibular first premolar distal surface, which is of great interest to adult patients not willing to show their appliance. To this end, the upper piece of the appliance should be placed on maxillary first molar distal surface. Should that be the case, if the patient has a small mouth, there is a high chance that the appliance will hurt patient’s cheek. A possible solution would be placing stops on the mesial surface of second molar tube (Fig 37), thus preventing the appliance from being distally placed in the dental arch and touching patient’s cheek. Hence, there is a great chance that the bracket will debond from mandibular first premolar. In order to prevent that from happening, it is advisable to have a bumper sleeve placed on its distal wing (Fig 38).

### Clinical considerations on PowerScope appliance

Full Class II correction treatment time might take from 6 to 12 months, with treatment time varying from patient to patient. In general, it is possible to claim that the appliance corrects molar relationship in 1mm each month. However, our clinical experience has shown that a brachyfacial adult patient reveals only 0.5-mm movement per month. Appliance installation takes about five minutes, while reactivation takes only 30 seconds. Debonding is also a quick procedure. Clinician should consider placing the 0.019 x 0.025-in stainless-steel wire at an appointment before PowerScope placement. Rectangular wire usually causes the patient to feel pain. As a result, he or she will associate such pain to PowerScope, not to the archwire. Allow PowerScope placement appointment to be as quick as possible.

## CLINICAL CASE 4 (Figs 39 to 43)

Patient aged 12 years at treatment onset. At the time, diagnosis was of Class II, division 2, left subdivision, with deep bite. Maxilla and mandible were well positioned. Lower facial height was decreased, with crowding in the mandibular anterior region. The patient presented with no skeletal deficiency; therefore, the option was for Class II correction with dentoalveolar changes provided by PowerScope appliance. Initially, fixed appliances were placed in both maxilla and mandible. After leveling, wire sequence reached 0.019 x 0.025-in stainless-steel wires placed in both maxilla and mandible. Subsequently, PowerScope appliance was installed and remained in place for four months, until overcorrected Class I molar relationship was achieved. After PowerScope, arch coordination and intercuspation began. The fixed appliance was then debonded, and retainers placed: maxillary and mandibular fixed 3x3 retainers, and maxillary Essix plate (Ace 0.040-in) used only during sleep. Treatment lasted for 17 months. 

**Figure 39:**
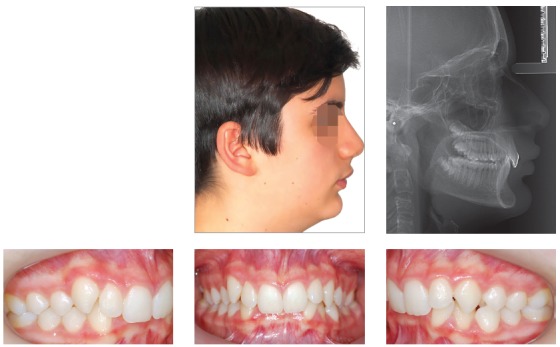
Pretreatment records: Extra and intraoral photographs, and lateral cephalogram.

**Figure 40:**
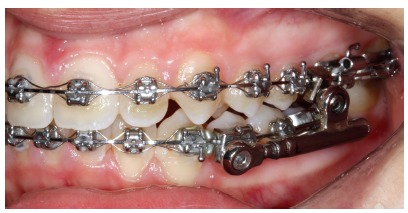
Intraoral left photograph showing PowerScope appliance placement.

**Figure 41:**
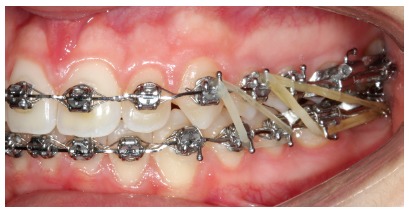
Intraoral left photograph showing fixed appliance at treatment completion.

**Figure 42:**
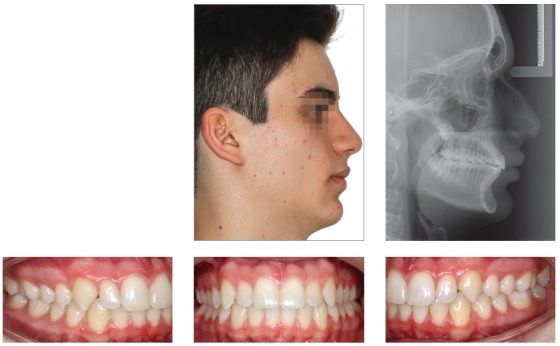
Posttreatment records: Extra and intraoral photographs, and lateral cephalogram.

**Figure 43:**
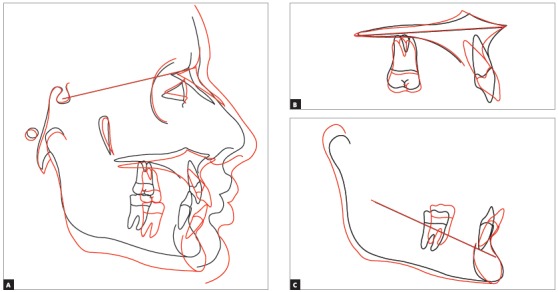
A) Cephalometric tracings superimposition on the cranial base (black = initial; red = treatment completion). B) Maxillary superimposition (ANS-PNS registered at ANS). C) Mandibular superimposition (Xi-Pm registered at Pm).

## INDICATIONS FOR FIXED FUNCTIONAL APPLIANCES USE

1. As Class II mechanics.
2. Cases of Class II with mandibular retrusion. Preference is given to rigid appliances.
2. Cases of Class II with maxillary protrusion.
3. Residual Class II correction after treatment with extractions.
4. Class II, subdivision, with no extraction treatment.
5. As anchorage after maxillary molars distalization.
6. As anchorage in cases with extractions.
7. As anchorage for space closure with mesialization of posterior teeth in cases of agenesis of mandibular second premolars or extraction of mandibular first molars.
8. Compensatory treatment of mandibular deficiency in adult patients.

## CONTRAINDICATIONS

There are some clinical situations in which the clinician needs to carry out cost-benefit analysis on the use of mandibular protraction appliances, namely:

1. Patients with periodontal issues.
2. Patients with thin gingiva in the mandibular anterior region.
3. Patients with mandibular incisors tipped or anteriorly projected.
4. Patients with marked gingival smile.
5. Patients with a tendency to open bite.

## HOW SHOULD FIXED FUNCTIONAL APPLIANCES BE USED IN CASES OF ASYMMETRICAL MALOCCLUSION?

Presently, there is a strong tendency towards treating Class II, subdivision, cases with fixed functional appliances.^44^ In those cases, the activated appliance will be actively placed on the Class II side. A non-activated appliance should always be placed on the Class I side, as it will help keeping the occlusal plane, in addition to guiding the mandible during closure. Should the appliance be placed on one side only, there is a great chance that it will lead to inclination of the occlusal plane. 

## COMPARISON BETWEEN CLASS II ELASTICS AND MANDIBULAR PROTRACTION APPLIANCES BIOMECHANICAL EFFECTS FOR CLASS II TREATMENT

**Figure 44:**
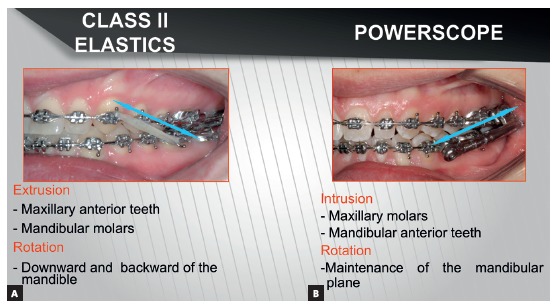
Direction of force for Class II correction: A) Class II elastics with traction force; B) Fixed functional appliance with impulsion force.

Because Class II elastics and fixed functional appliances are both used to treat Class II malocclusion, a number of clinicians believe they are the same thing. However, they are not. In terms of force, elastics perform intermittent action, while fixed functional appliances perform continuous action. Elastics exert traction, while fixed functional appliances exert impulsion (Fig 44). Vertical component of traction might extrude maxillary incisors and mandibular molars as a result of elastics use. Consequently, effect on the occlusal plane is clockwise rotation, with resulting downward and backward mandibular rotation.^45^ In Class II dolichofacial patients with increased mandibular plane angle, the mechanics tending to extrude posterior teeth is not recommended. Fixed functional appliances use impulsion over the occlusal plane, that is, they push while separating appliance insertion points. Force is intrusive in both maxillary buccal segment and mandibular anterior segment; as a result, the effect of occlusal plane rotation decreases.^45^ Therefore, the tendency is towards keeping mandibular plane inclination.^46^ This mechanics might be beneficial to treat vertical-pattern patients, as well as in deep bite cases. As for patients with a tendency towards open bite, care should be taken regarding the use of fixed functional appliances. Mini-implants could also be used in vertical-pattern patients, if placed in the buccal segment, with a view to enhancing the effect of molar intrusion produced by mandibular protraction appliances. As a result, real counterclockwise mandibular rotation^47^ and greater chin advancement would be produced.

**Figure 45:**
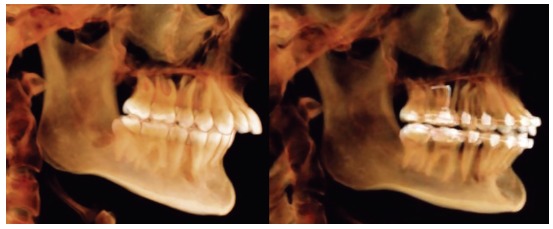
Class II compensatory treatment with PowerScope 2 in adult patient not willing to undergo orthognathic surgery.

## CLASS II COMPENSATORY TREATMENT

Many adult patients with mandibular retrusion and recommendation for surgical treatment prefer not to undergo orthognathic surgery. In those cases, the possibility of compensatory treatment is considered,^48^ whether with maxillary premolars extraction or use of fixed functional appliances. Maxillary premolars extraction with retraction of maxillary incisors has great chances of resulting in profile flattening,^48^ especially in cases with normal nasolabial angle and increased overjet. Should that be the case, preference is given to fixed functional appliance, as it will result in little distalization of maxillary teeth and mesialization of mandibular teeth (Fig 45).

## CONCLUSIONS

1. Rigid fixed functional appliances provide better skeletal results than flexible and hybrid ones.
2. Flexible and hybrid appliances have similar effects to those produced by Class II elastics. They ultimately correct Class II with dentoalveolar changes.
3. From a biomechanical standpoint, fixed functional appliances are more recommended to treat Class II in dolichofacial patients, in comparison to Class II elastics.
